# Ocrelizumab‐induced colitis: VigiBase disproportionality analysis, case reports and literature review

**DOI:** 10.1002/bcp.70490

**Published:** 2026-02-15

**Authors:** Audrey Fresse, Christophe Hommel, Nadine Petitpain, Philippe Kerschen

**Affiliations:** ^1^ Regional Pharmacovigilance Center of Nancy, Pharmaco‐toxicology Department University Hospital of Nancy Vandoeuvre‐lès‐Nancy France; ^2^ Department of Gastroenterology Centre Hospitalier Emile Mayrisch Esch sur Alzette Luxembourg; ^3^ Department of Neurology Centre Hospitalier Luxembourg Luxembourg‐Ville Luxembourg

**Keywords:** adverse drug reaction, colitis, disproportionality analysis, ocrelizumab, pharmacovigilance

## Abstract

**Aims:**

Ocrelizumab is a humanized anti‐CD20 monoclonal antibody used in multiple sclerosis. Since its commercialization, several cases of ocrelizumab‐induced colitis have been reported in the scientific literature.

**Methods:**

To explore the potential association of ocrelizumab with colitis as an adverse drug reaction (ADR), we conducted a descriptive and disproportionality analysis of ocrelizumab‐induced colitis registered in VigiBase, as well as an extensive literature review and a description of two cases from Luxembourg reported to our pharmacovigilance centre.

**Results:**

Our VigiBase analysis revealed 400 cases of ocrelizumab‐induced colitis, with 79.0% serious cases and a median time to onset of 17 months. The preferred term (PT) coded was ‘colitis’ (53.0%), followed by PTs related to inflammatory bowel diseases (33.5%). Significant disproportionality was found for high‐level term ‘colitis’ and for ‘colitis’, ‘colitis microscopic’ and ‘immune‐mediated enterocolitis’ PTs. Our literature review revealed 24 case reports corresponding to 36 patients with a median time to onset of 18 months and a predominance of unspecified colitis (61.1%) and inflammatory bowel diseases (27.8%). Two patients relapsed after ocrelizumab resumption.

**Conclusions:**

Together, our findings of two relevant clinical cases of ocrelizumab‐induced colitis, the literature review and the significant VigiBase disproportionality, suggest a potential safety signal for ocrelizumab.

1 **What is already known about this subject**

- Data concerning colitis with anti‐CD20 monoclonal antibodies, including ocrelizumab, began to emerge.
- Risk of ocrelizumab's immune‐mediated colitis had been recognized by the FDA and Swiss medical agency but is still not disclosed in the British and European SmPC.

**What this study adds**

- Together, our findings of two relevant clinical cases of ocrelizumab‐induced colitis, the literature review and the significant VigiBase disproportionality, suggest a potential safety signal for ocrelizumab and colitis.

## 1 INTRODUCTION

Ocrelizumab is a humanized anti‐CD20 monoclonal antibody commercialized in Europe since 2018 for multiple sclerosis (MS). It acts by selectively depleting B lymphocytes, and it is similar to rituximab (89% homology). Ocrelizumab is particularly commonly used for patients with relapsing‐remitting MS (RRMS) and primary progressive MS (PPMS). Owing to its rather benign adverse drug reaction (ADR) profile (mainly infusion reactions, lymphopenia, neutropenia and respiratory infection)^1^ and its effectiveness on MS, prescription of ocrelizumab is increasing. However, data concerning immune‐mediated ADRs have begun to emerge concerning the use of anti‐CD20 monoclonal antibodies, including ocrelizumab.^2^

Immune colitis is well known to be associated with MS, as inflammatory bowel disease (IBD) is reported in 0.6% of patients with MS, and MS is reported in 0.2% of patients with IBD.^3^ However, some studies have shown an increased risk for colitis in MS patients treated with rituximab^4^ and in patients receiving rituximab for other indications.^5^, ^6^ Moreover, since 2020, several clinical case reports of ocrelizumab‐induced colitis with compatible chronologies have been published,^4^, ^7^, ^8^, ^9^, ^10^, ^11^, ^12^, ^13^, ^14^, ^15^, ^16^, ^17^, ^18^, ^19^, ^20^, ^21^, ^22^, ^23^, ^24^, ^25^, ^26^, ^27^, ^28^ and a signal was detected by the Food and Drug Administration (FDA) Advanced Reporting System (FAERS).^29^, ^30^ This led the USA FDA to update the Summary of Product Characteristics (SmPC) of ocrelizumab in August 2022 by adding a warning about immune‐mediated colitis.^31^ A similar update was made in October 2023 by the Swiss medical agency.^32^ Nevertheless, the causal role of ocrelizumab in colitis in patients treated for MS is still unclear,^33^ and colitis is currently not disclosed in the European SmPC.

Disproportionality analysis is an analysis method for pharmacovigilance databases that has been used since the 1990s and more widely since the late 2010s. Although it does not provide a clear measure of the risk linked to a specific ADR, such as in clinical trials or pharmacoepidemiological studies, due to some inherent limitations of pharmacovigilance databases,^34^ it can help define potential pharmacovigilance signals. These signals could be later confirmed or invalidated by a detailed analysis of pharmacovigilance cases and/or the realization of pharmacoepidemiological studies.

Given that we recently received two reports from Luxembourg of ocrelizumab‐induced *de novo* colitis to our regional pharmacovigilance centre, we decided to conduct a descriptive and disproportionality analysis of ocrelizumab induced‐colitis registered in VigiBase to explore this potential drug–ADR link and to complete clinical data with an extensive literature review.

## 2 METHODS

### 2.1 VigiBase analysis

On 24 February 2025, we extracted from the WHO pharmacovigilance database (VigiBase) all individual case safety reports (ICSRs) registered with ocrelizumab as a suspect or interacting drug and at least one ADR corresponding to the MedDRA high‐level term (HLT) ‘colitis (excl infective)’. We deliberately chose a large term to ensure that all the colitis ICSRs, including those that may have been incorrectly coded, were extracted. We analysed all the available information. Cases were considered ‘well documented’ if their completeness score was equal to or above 0.8.^35^ Cases were regrouped under the term ‘inflammatory bowel disease’ (IBD) if they included any of the following preferred terms (PTs): ‘Crohn's disease’, ‘colitis ulcerative’, ‘acute haemorrhagic ulcerative colitis’ and/or ‘inflammatory bowel disease’. The outcome was considered not assessable if information was either lacking on dechallenge or reaction resolution. Rechallenge was considered not assessable if information was lacking either on rechallenge or reaction recurrence. An outcome was considered favourable if the patient was either recovering or recovered (with or without sequelae). The outcome was unfavourable if the patient had not recovered by the time the ICSR was registered or if the patient died because of colitis.

Disproportionality analysis of VigiBase was performed via two different methods, reporting the odds ratio (ROR), which offers a more sensitive approach, and the information component (IC), which is more specific. Considering the following contingency table (see Table 1 for letters' correspondence), calculations were performed as presented below.

**TABLE 1:** Contingency table.

	Reports with ADR of interest	Reports with ADR of interest
Reports with ocrelizumab	a	b
Reports without ocrelizumab	c	d

The ROR was calculated as $\mathrm{ROR}=\frac{a*d}{b*c}$ with a 95% confidence interval (CI) corresponding to $\mathrm{ROR}\times e^{\pm 1.96\sqrt{\frac{1}{a}+\frac{1}{b}+\frac{1}{c}+\frac{1}{d}}}$. The ROR was significant if the CI did not include 1. If the CI's lower limit was greater than 1, then the drug‐ADR pair was considered significantly more frequently reported with that drug than with other drugs. Notably, the ROR was only applicable if at least five ADRs of interest were reported with the suspected drug.

The IC was calculated as $\mathrm{IC}=\frac{N_{\text{observed}}+0.5}{N_{\text{expected}}+0.5}\;\times \;\log2$ with a 95% CI corresponding to ${\mathrm{IC}}_{025}=\mathrm{IC}-\frac{3.3}{\sqrt{N_{\text{observed}}+0.5}}-\frac{2}{\sqrt[{3}]{N_{\text{observed}}+0.5}}$ and ${\mathrm{IC}}_{975}=\mathrm{IC}-\frac{2.4}{\sqrt{N_{\text{observed}}+0.5}}-\frac{0.5}{\sqrt[{3}]{N_{\text{observed}}+0.5}}$, where $N_{\text{expected}}=\frac{N_{\text{substance}}\;\times \;N_{\text{reaction}}}{N_{\text{total}}}$. It was interpreted in relation to 0, in the same way as the ROR.

To allow a more precise analysis, disproportionality analysis was performed on HLT and on all PTs included in it. We did not perform a sensitivity analysis by removing ICSRs that also suspected other drugs known to induce colitis due to the low number of ICSRs suspecting another drug.

### 2.2 Literature review

On 24 February 2025, we queried the PubMed, Reactions Weekly and Google Scholar databases for all case reports related to ocrelizumab and colitis. All identified articles in English and French, as well as their bibliography, were screened and added to the review if deemed relevant.

### 2.3 Statistics

Qualitative data are expressed as percentages. Quantitative data are expressed as the mean ± standard deviation (median; min–max). Disproportionality analyses are described in the relevant section. All the statistical analyses were performed by Excel®. Fisher's test was used to compare proportions due to the small number of cases for some parameters (<5).

## 3 RESULTS

### 3.1 Case reports

#### 3.1.1 Patient 1

The treatment of a 62‐year‐old woman with ocrelizumab 600 mg/6 months was initiated in January 2022 for PPMS. She had a history of hypercholesterolemia; arterial hypertension treated with candesartan; valvular heart disease; hemochromatosis; gastroesophageal reflux disease treated with esomeprazole 20 mg/day; hiatal hernia; sigmoidal diverticulosis in 2021; cholecystectomy in June 2022; hypothyroidism treated with levothyroxine; depressive syndrome treated with sertraline and alprazolam; and supplementation with folic acid. She had no allergies, did not smoke and did not consume alcohol.

In February 2022, shortly after ocrelizumab initiation, the patient presented with nausea, abdominal pain and chronic diarrhoea. Initially, a link with ocrelizumab seemed unlikely, and the treatment was continued. In October 2022, because of persistent symptoms after the third infusion of ocrelizumab, an abdominal computed tomography (CT) was performed and revealed colitis involving the ascending, transverse and sigmoid colon. No colonoscopy was performed at the time. Oral antibiotics did not improve symptoms. Ocrelizumab was discontinued.

In May 2024, due to persistent symptoms with weight loss of 10 kg, the patient was hospitalized for chronic diarrhoea, abdominal pain and an altered general condition. At the time of hospitalization, the patient was not receiving any ongoing treatment for MS, in accordance with her request, despite MS progression. CT revealed pancolitis. She had mild hypokalaemia (3.2 mM), hypocalcaemia (2.1 mM), hypoalbuminemia (33 g/L), an increased lipase level (4 ULN) and a slightly elevated C‐reactive protein level (8 mg/L). The faecal calprotectin concentration was 80 μg/g (normal value < 50 μg/g), whereas the stool culture was negative. Other blood, renal, hepatic and thyroid tests were normal, with no vitamin or iron deficiencies identified. Rectosigmoidoscopy revealed oedematous rectosigmoiditis with large folds without ulcers. Biopsies revealed acute ulcerated colitis associated with glandular atypia without dysplasia, which is compatible with drug‐induced colitis. Colonoscopy revealed colitis, with hyperplastic and inflammatory features, without ulcers. Pancolitis spared the right colon and some parts of the transverse colon.

The patient was treated with intravenous methylprednisolone (2 mg/kg/day) and mesalamine (3 g/day), with total regression of diarrhoea at the end of hospitalization. Owing to malnutrition and ionic disorders, she also received temporary parenteral nutrition and ionic supplementation. The patient was discharged within 2 weeks and received oral degressive corticosteroid and mesalamine treatments.

#### 3.1.2 Patient 2

A 56‐year‐old male patient received ocrelizumab 600 mg for 9 months since November 2022 for PPMS. His medical history included arterial hypertension treated with amlodipine and benign prostatic hyperplasia treated with tamsulosin.

In November 2024, 5 months after his third ocrelizumab infusion, the patient presented with diarrhoea with mucous stools, abdominal cramps and tenesmus. Colonoscopy led to the diagnosis of indeterminate left‐sided colitis (patchy erosions and erythematous mucosa with signs of chronicity and scarring in the sigmoid and rectum), whereas an infectious workup (sexually transmitted diseases, bacterial and parasitic) was negative. Histopathology revealed signs of subacute colitis with distorted crypt architecture, cryptitis and microabscesses but no granulomas. The outcome was progressively favourable under mesalamine, loperamide, racecadotril, azithromycin (initiated by the primary care physician before definitive results), phloroglucinol and metoclopramide. A subsequent ocrelizumab infusion was administered in March 2025, but digestive symptoms recurred a few days later. The abdominal scanner revealed extended pancolitis, excluding toxic megacolon or pneumatosis. Stool analysis for infectious aetiologies, including the *Clostridium difficile* test, was negative. A mild biological inflammatory syndrome was also present, disappearing rapidly under empirical antibiotics (metronidazole). However, as the patient's digestive symptoms persisted, a new colonoscopy was performed, which revealed similar endoscopic and histopathological findings, particularly cytomegalovirus‐negative immunostains. Methylprednisolone and mesalamine were initiated in April 2024, leading to rapid clinical remission, but corticosteroid dependence occurred 2 months later, with suspicion of corticosteroid resistance. At that time, the patient was on vedolizumab therapy. Follow‐up endoscopy was planned after 3 months to assess the endoscopic and histologic response.

### 3.2 Literature review

Our literature review retrieved 24 published case reports corresponding to 36 patients, which were reviewed as described in Table S1.^4^, ^7^, ^8^, ^9^, ^10^, ^11^, ^12^, ^13^, ^14^, ^15^, ^16^, ^17^, ^18^, ^19^, ^20^, ^21^, ^22^, ^23^, ^24^, ^25^, ^26^, ^27^, ^28^, ^36^ Patients were predominantly females (*n* = 28, 78%), with an average age of 42 ± 13 years (40; 24–68). Four patients had a previous gastrointestinal history before colitis (ileal perforation with small bowel resection, Crohn's disease, coeliac disease, chronic gastritis). Colitis occurred 599 ± 433 days (549; 7 days–5 years) after the start of ocrelizumab, including three cases that occurred after the first dose (8.3%).^7^, ^8^, ^24^ The diagnosis was IBD in 10 patients (27.8%, including a relapse of preexisting Crohn's disease), microscopic colitis in three patients (8.3%) and typhlitis in one patient (2.8%). In the other patients (22, 61.1%), the type of colitis was unspecified. The final recorded diagnosis corresponds to the diagnosis cited by the authors (Table S1). However, considering the accumulation of data concerning ocrelizumab‐induced colitis, the terms ‘medication‐associated colitis’ or ‘ocrelizumab‐associated colitis’ could be relevant for these cases. The diagnosis profile did not differ according to TTO, as three out of 11 patients (27.3%) presented with an IBD within their first year of treatment, whereas six out of 21 patients (28.6%) presented with an IBD after 1 year of treatment (*p* = 1, unknown TTO for four patients, including one IBD). Two patients^26^, ^27^ experienced recurring digestive symptoms after each infusion. With respect to colitis management, 27 patients (75.0%) required corticosteroids. Other coadministered treatments were either specific (mesalamine, monoclonal antibodies, intestinal anti‐inflammatory drugs, immunosuppressive drugs and/or surgery), symptomatic (5, 13.8%), or antimicrobial (4, 11.1%). Ocrelizumab was stopped in 23 patients (63.9%), with favourable outcomes in 19 patients, no recovery in two patients and unknown outcomes in two patients. Ocrelizumab was continued in seven patients (19.4%) in association with specific or symptomatic treatment, and the outcome was favourable for all patients. In 11 patients (30.6%), ocrelizumab was switched to another treatment without colitis relapse, including a switch to ofatumumab without recurrence after 4 months of follow‐up. Information on ocrelizumab continuation was unavailable for six patients (16.7%).

### 3.3 VigiBase disproportionality analysis

Our VigiBase extraction retrieved 400 cases of colitis associated with ocrelizumab that were registered in VigiBase. The patients' characteristics are presented in Table 2. Notably, 54 cases were ‘reports from studies’, but no duplicates within our literature review were identified on the basis of information available in VigiBase, which was subject to the underfilling of ‘study names’ (21 cases not filled or unknown). Almost all the cases involved adults, with the exception of one case reported in a neonate after foetal ocrelizumab exposure. Patients were mostly women (274, 68.5%) treated for MS (315, 78.7%). The cases were mainly serious (316, 79.0%), with ‘caused/prolonged hospitalization’ being the most reported seriousness criterion. The most reported PT was ‘colitis’ (212, 53.0%), followed by PTs related to an IBD (134, 33.5%). The median time to onset (TTO) was 17 months. Other drugs were cosuspected in 43 patients (10.7%). The most common cosuspected drug was rituximab (2.8%). With respect to dechallenge, ocrelizumab was discontinued in 10.0% of patients, mostly those with favourable outcomes (67.5%). For patients in whom ocrelizumab was continued, outcomes were more common, as 48.3% had a favourable outcome and 51.7% an unfavourable outcome. With respect to the seven ICSRs with ‘death’ severity criteria, colitis was considered the cause of death in three patients, including one patient with concomitant sepsis and one patient with concomitant COVID‐19 pneumonia‐induced respiratory failure and anaemia. In one fatal case, the outcome for colitis was unknown in the context of complicated COVID‐19 pneumonia with acute respiratory failure, emphysema, pneumothorax and septic shock. The three patients had favourable outcomes for their colitis at the time of their death from another cause (one with cardiac failure, one with progressive multifocal leukoencephalopathy and one with an unspecified cause). Rechallenge was mentioned in only two cases: one with reaction recurrence and the other without.

**TABLE 2:** Characteristics of ocrelizumab‐induced colitis cases reported in VigiBase.

	*N* (%)/mean ± standard deviation (median; min–max)
Countries	
American countries	282 (70.5%)
European countries	89 (22.2%)
Oceanian countries	22 (5.5%)
African countries	4 (1.0%)
Asian countries	3 (0.8%)
Completeness score	0.40 ± 0.24 (0.33; 0.10–1.00)
Well‐documented cases	45 (22.5%)
Age (years)	48 ± 14 (49; 14 days–82 years)
	160 unknown (40.0%)
Sex	
Female	274 (68.5%)
Male	65 (16.3%)
Unknown	61 (15.3%)
Ocrelizumab's indication	
*Multiple sclerosis (MS)*	315 (78.7%)
MS without precision	135 (33.7%)
Primary progressive MS	33 (8.2%)
Progressive relapsing MS	1 (0.2%)
Relapsing MS	135 (33.7%)
Secondary progressive MS	11 (2.7%)
*Arteritis*	1 (0.2%)
*Ulcerative colitis*	1 (0.2%)
*Unknown nervous system disorder*	1 (0.2%)
*Unknown indication*	83 (20.7%)
Reported adverse drug reaction according to preferred term	
Colitis	212 (53.0%)
Crohn's disease	76 (19.0%)
Colitis ulcerative	44 (11.0%)
Colitis microscopic	40 (10.0%)
Immune‐mediated enterocolitis	14 (3.5%)
Inflammatory bowel disease	13 (3.3%)
Colitis ischaemic	10 (2.5%)
Necrotising colitis	3 (0.8%)
Neutropenic colitis	3 (0.8%)
Autoimmune colitis	3 (0.8%)
Terminal ileitis	2 (0.5%)
Eosinophilic colitis	1 (0.3%)
Acute haemorrhagic ulcerative colitis	1 (0.3%)
Serious	
No	84 (21.0%)
Yes	316 (79.0%)
*Seriousness criteria*	
Death	7 (1.8%)
Life threatening	4 (1.0%)
Caused/prolonged hospitalization	140 (35.0%)
Disabling/incapacitating	10 (2.5%)
Other medically important conditions	205 (51.3%)
Time to onset (months)	19 ± 16 (17; 3 days–5 years)
	349 unknown (87.2%)
Dechallenge	
*Ocrelizumab stopped*	40 (10.0%)
Favourable outcome	27 (67.5%)
Unfavourable outcome	13 (32.5%)
*Ocrelizumab continued*	29 (7.2%)
Favourable outcome	14 (48.3%)
Unfavourable outcome	15 (51.7%)
*Unknown/not assessable*	331 (82.7%)
Other coreported suspected drugs	43 cases (10.7%)
Rituximab	11 (2.8%)
Diazepam	3 (0.8%)
Natalizumab	3 (0.8%)
Tozinameran	3 (0.8%)
Methylprednisolone	2 (0.5%)
Cortisone	2 (0.5%)
Dimethyl fumarate	2 (0.5%)
Ofatumumab	2 (0.5%)
Other suspected drug (one case each)	21 (5.2%)

*Note*: American countries: United States of America, Canada and Mexico; European countries: Germany, Switzerland, Spain, United Kingdom, Italy, France, Belgium, Hungary, Romania, Netherlands, Czechia, Greece, Croatia, Ireland, Luxembourg and Portugal; Oceanian countries: Australia and New Zealand; African country: Egypt; Asian country: United Arab Emirates. Other coreported suspected drugs: alemtuzumab, amlodipine, amoxicillin, budesonide, clonazepam, diclofenac, elasomeran, escitalopram, fingolimod, furosemide, gabapentin, glatiramer, infliximab, interferon β‐1a, interferon β‐1b, pembrolizumab, placebo, pregabalin, teriflunomide, ublituximab, vedolizumab.

The disproportionality analysis results are presented in Table 3. Significant disproportionality was found for HLT ‘colitis’, as well as for PT ‘colitis’, ‘colitis microscopic’ and ‘immune‐mediated enterocolitis’, for both disproportionality methods.

**TABLE 3:** Vigibase's disproportionality analysis of ocrelizumab‐induced colitis.

	ROR (CI)	IC (CI)
HLT colitis (excl infective)	**2.36 (2.14–2.61)**	**1.23 (1.07–1.40)**
PT colitis	**4.55 (3.98–5.21)**	**2.17 (1.94–2.40)**
PT Crohn's disease	1.22 (0.98–1.53)	0.29 (−0.09–0.67)
PT colitis ulcerative	1.13 (0.84–1.52)	0.18 (−0.32–0.68)
PT colitis microscopic	**8.86 (6.49–12.10)**	**3.00 (2.47–3.53)**
PT immune‐mediated enterocolitis	**3.43 (2.03–5.79)**	**1.66 (0.75–2.56)**
PT inflammatory bowel disease	1.24 (0.72–2.13)	0.30 (−0.64–1.23)
PT colitis ischaemic	1.41 (0.76–2.63)	0.47 (−0.61–1.55)
PT necrotising colitis	NA	0.99 (−1.08–3.06)
PT neutropenic colitis	NA	0.88 (−1.19–2.95)
PT autoimmune colitis	NA	1.31 (−0.76–3.38)
PT terminal ileitis	NA	1.42 (−1.18–4.01)
PT eosinophilic colitis	NA	0.96 (−2.82–4.75)
PT acute haemorrhagic ulcerative colitis	NA	1.34 (−2.45–5.12)

Abbreviations: CI: confidence interval, HLT: high‐level term, IC: information component, NA: not applicable, PT: preferred term, ROR: reported odds ratio.

## 4 DISCUSSION

To our knowledge, this is the first study on ocrelizumab‐induced colitis that focused on VigiBase data and included clinical data from clinical cases and an extensive scientific literature review. We found significant disproportionality for ocrelizumab and colitis, including microscopic colitis, which was in line with the findings in the literature.

Although the specific pharmacological mechanism remains unknown, ocrelizumab‐induced colitis is presumed to be caused by the depletion of regulatory B lymphocytes, thus leading to a proinflammatory state in the colon.^37^ Interestingly, cases of colitis have also been reported with other anti‐CD20 drugs, such as rituximab, ofatumumab and obinutuzumab,^30^, ^38^, ^39^ suggesting a class effect, with morbidity and mortality rates higher than those of other colitis‐inducing drugs. In our VigiBase data, ublituximab, another anti‐CD20 monoclonal antibody, was also cosuspected in one patient. However, chronological data were lacking for this case and did not allow us to draw conclusions for a possible case of cross‐reactivity. We also noted three reported cases of diverticulitis following ocrelizumab,^40^, ^41^ which suggests possible more widespread gastrointestinal toxicity, including infectious causes such as cytomegalovirus infection.^21^

Our VigiBase analysis and published case reports are clinically similar in terms of age, female predominance, TTO and the occurrence of IBD (33.5% *vs*. 27.8%) and microscopic colitis (10.0% *vs*. 8.3%). These findings are also consistent with the FAERS data analysis by Kim et al.^29^ regarding patient demographics, seriousness and colitis type. Patient management was also similar to that of patients receiving corticosteroid therapy as the first‐line treatment. However, the median TTO was shorter in the FAERS group (8 months *vs*. 17–18 months in our study). This difference might be explained by different populations or by the important number of missing data in our VigiBase study. Nevertheless, our TTO is consistent with the TTO reported for obinutuzumab.^38^ The persistence of B lymphocyte depletion for up to 18 months after the last dose of ocrelizumab was administered could explain the delayed TTO in some cases.^42^ Colitis may develop faster in predisposed patients, as demonstrated by the three cases reported after the first ocrelizumab dose,^7^, ^8^, ^24^ and in our first patient.

An important discussion point is the possible link between IBD and MS itself. MS patients have a higher incidence of IBD than control subjects do, and vice versa.^3^ Thus, it would be tempting to attribute the onset of colitis to this increased risk of IBD among MS patients rather than to ocrelizumab. However, several points should be considered. First, fewer than one‐third of reported cases of colitis (27.8%–33.5%) were diagnosed with IBD. Second, we observed relevant chronological arguments for the involvement of ocrelizumab, such as the occurrence of colitis within days or weeks after ocrelizumab infusion, as well as our reported cases of positive rechallenge and shorter TTO after ocrelizumab resumption. Third, Coletta and Rupawala reported one case of Crohn's disease aggravation after ocrelizumab initiation in a patient who was stable before treatment and a similar case after rituximab.^17^ Finally, colitis has also been described with other anti‐CD20 drugs in non‐MS patients, which strengthens the role of these drugs in patients developing colitis and suggests a class effect.^5^, ^43^

The exact contribution of the B‐cell compartment to the pathophysiology of colitis remains largely unknown. Rituximab has been shown to be ineffective in the treatment of ulcerative colitis in a small Phase 2 trial, although it did not worsen disease activity either.^44^ Although plasmablasts (a mostly CD19+ CD20− cell type) have recently been implicated in the pathophysiology of ulcerative colitis,^45^ real‐life use of CD19 targeting therapies has yielded conflicting effects. Several cases of colitis are already reported after CD19‐targeting CAR T‐cell therapy for B‐cell lymphoma.^46^, ^47^, ^48^, ^49^ On the other hand, a recent case‐report relates the use with success of a CD19 CAR‐T cell in a multiresistant ulcerative colitis.^50^ This illustrates the lack of knowledge about the involvement of specific B‐cell subpopulations in different colitis phenotypes. It also highlights the need for further studies, especially in the context of the growing use of anti‐CD20 and anti‐CD19 therapies, whether by means of monoclonal antibodies, bispecific T cell engagers or CAR‐T cells.

Our study had several limitations. The main limitation was lacking information on some parameters in both VigiBase and some cases reported in the literature. VigiBase in particular had limited information about patients' medical history, and preexisting IBD relapse was difficult to detect. In the disproportionality analyses, we chose to keep cases with cosuspected drugs known to induce colitis (such as pembrolizumab or infliximab) or drugs with a low probability of inducing colitis (such as placebo or drugs used to treat colitis) owing to their small number and the impossibility of accessing the cases' narrative to assess the imputability of these drugs. The lack of chronological data might also have affected the median TTO calculation, which could explain the differences we observed with FAERS data,^29^ as well as the outcome regarding dechallenge. The inability to access the cases' narratives also prevented us from identifying any coding errors regarding the final diagnosis of colitis. We cannot exclude either the possibility of duplicates between the cases retrieved from our VigiBase analysis and those from the literature review, despite checking the VigiBase data, due to missing information on the study name. It is also important to note that no ADR frequency can be calculated from VigiBase, as it only relies on spontaneous reports. Finally, underreporting is a major problem of pharmacovigilance databases, going up to 95% of unreported ADRs,^51^ and also questioned the type of cases being reported in those databases, as some reporting biases can be observed after commercialization of a new drug or mediatization of an ADR.

Our reported findings including two relevant clinical cases of ocrelizumab‐induced colitis, a literature review and the significant disproportionality in VigiBase suggest a potential safety signal associated with ocrelizumab. Considering that other anti‐CD20 agents are also associated with this risk, even in non‐MS patients, drug‐induced colitis might be considered a class effect. This potential signal needs to be confirmed by clinical and/or pharmacoepidemiological studies, especially regarding the possibility of continuing ocrelizumab treatment under corticosteroids or not, or replacing it with another anti‐CD20 drug. However, until these data become available, we invite practitioners to carefully monitor patients who report digestive symptoms during ocrelizumab therapy and to not overlook possible immune‐mediated colitis, especially regarding the severity of this condition.

## AUTHOR CONTRIBUTIONS

**Audrey Fresse:** Conceptualization, data curation, formal analysis, investigation, methodology, resources, writing—original draft preparation, writing—review and editing. **Christophe Hommel:** Investigation, resources, writing—review and editing. **Nadine Petitpain:** Conceptualization, data curation, formal analysis, investigation, methodology, resources, writing—original draft preparation, writing—review and editing. **Philippe Kerschen:** Investigation, resources, writing—review and editing. All authors had full access to all the data in the study and had final responsibility for the decision to submit for publication.

## CONFLICT OF INTEREST STATEMENT

The authors declare no conflicts of interest.

## Supporting information


**Table S1:** Description of literature review on ocrelizumab‐induced colitis.

## Data Availability

The data are available on reasonable demand from Dr. Audrey FRESSE.
