# Supplementary material for: Ocrelizumab‐induced colitis: VigiBase disproportionality analysis, case reports and literature review
Source: Br J Clin Pharmacol. 2026 Feb 15;92(7):2162–70. doi: 10.1002/bcp.70490 (PMC13304271; doi:10.1002/bcp.70490)
Supplement: Supplementary file 1 — Table S1: Description of literature review on ocrelizumab‐induced colitis. [file BCP-92-2162-s001.docx]

Supplementary table 1: Description of literature review on ocrelizumab induced colitis

| Article | Patient (y.o.) | Medical history | Previous MS treatment | Concomitant medicines | Clinical presentation | TTO (TTO from last dose if known) | Biopsy | Biological data | Final diagnosis | Treatment | Decision regarding ocrelizumab | Outcome |
| --- | --- | --- | --- | --- | --- | --- | --- | --- | --- | --- | --- | --- |
| Akram 2020 | ♂ 62 | PPMS | Corticosteroids, IFN β1a, DMF | UNK | Non bloody diarrhoea, dehydration, weight loss | 1 week | Moderately active colitis with mild architectural changes and mild plasmacytosis. Immunohistochemistry showed abundant CD3 cells with paucity of CD19 and CD 20 cells. biopsies showed thickened subepithelial collagen layer typical of collagenous colitis | Calprotectin ↑, ESR ↑, CRP ↑ | Pancolitis | Methylprednisolone, prednisone, budesonide | Stopped | Recovering but colitis progressing to collagen colitis |
| Aljaberi 2023 | ♂ 26 | RRMS | UNK | UNK | UNK | 4 years | Inflammatory bowel disease | UNK | IBD | UNK | Stopped | UNK |
| Au 2022 | ♀ 45 | MS, JCV infection | Fingolimod, natalizumab | No | Diarrhoea | 5 years | Patchy mild active inflammation | Calprotectin ↑ | Crohn disease | Vedolizumab, azathioprine | Continued | Favourable |
| Barnes 2021 | ♀ 56 | PPMS | UNK | UNK | Bloody diarrhoea, odynophagia, general malaise, fever | 18 months | Patchy, moderately-active chronic inflammation with cryptitis and crypt abscess formation with architectural disturbance, without granulomas. No CD20+ cells on immunohistochemistry. | CRP ↑ | Crohn disease | Hydrocortisone, prednisolone, pantoprazole, sucralfate | Stopped | Recovering but colitis progressing to microscopic colitis |
| Carballo-Folgoso 2022 | ♀ 29 | RRMS, JCV infection | Fingolimod, natalizumab | UNK | Loose watery stools | 18 months | Severe active inflammation with cryptitis, crypt abscess formation with architectural disturbance and the presence of granulomas, suggestive of Crohn's disease | UNK | Crohn disease | Prednisone, ustekinumab | Continued | Favourable |
| Challa 2024 | ♀ 46 | MS | UNK | UNK | Abdominal pain, diarrhoea, fever | 6 months | Initial: chronic active colitis/proctitis. Follow-up: focal acute colitis. | UNK | Colitis | Prednisone, mesalamine | Stopped | Favourable |
|  | ♀ 48 | MS | UNK | Fingolimod, natalizumab | Watery diarrhoea | 15 months (3 months) | Focal acute colitis | UNK | Colitis | Loperamide | Continued | Favourable |
|  | ♀ 30 | MS | UNK | UNK | Bloody diarrhoea, hematochezia | 21 months (3 months) | Initial: acute colitis/proctitis. Follow-up: apoptotic colopathy | UNK | Colitis | Prednisone | Stopped | Favourable |
|  | ♀ 24 | MS | UNK | Ibuprofen | Hematochezia, weight loss | 32 months (2 months) | Acute colitis | UNK | Colitis | UNK | Continued | Favourable |
|  | ♂ 68 | MS | UNK | Metformin | Watery diarrhoea | 30 months (6 months) | Initial: focal acute colitis. Follow-up: lymphocytic colitis | UNK | Lymphocytic colitis | Budesonide, loperamide | Continued | Favourable |
|  | ♀ 31 | MS | UNK | UNK | Abdominal pain, diarrhoea | 13 months (7 months) | Initial: acute colitis and proctitis. Follow-up: normal mucosa | UNK | Colitis | Prednisone, mesalamine, sulfasalazine | Stopped | Favourable |
| Chatto 2025 | ♀ 60 | PPMS | UNK | UNK | Prolonged diarrhoea | 2 months | Lymphocytic colitis | UNK | Microscopic colitis | Budesonide | Continued at half-dose | Favourable |
| Chowdhury 2024 | ♀ 47 | MS, GERD, AH, PH, PCOS, migraine | UNK | Omeprazole, galcanezumab, lisinopril, metoprolol, metformin | Post-cholecystectomy epigastric pain, nausea, vomiting, fever | 18 months | UNK | UNK | Localized colitis | Corticosteroids | Continued | Favourable |
| Colletta 2023 | ♀ 54 | MS, ileal perforation, small bowel resection | UNK | UNK | Severe diarrhoea, hematochezia | 3 years | Active enterocolitis with pathology positive for crypt architectural disarray | UNK | Crohn disease | Prednisone | Stopped | Not recovered |
|  | ♂ 51 | MS, ankylosing spondylitis, Crohn disease | UNK | 5-ASA | UNK | 1 year | UNK | Calprotectin ↑ | Crohn disease | UNK | Stopped | Not recovered |
| Cook 2023 | ♀ 31 | MS | UNK | UNK | Bloody diarrhoea, intense abdominal cramping | 2 years (1 week) | Benign biopsy | UNK | Severe colitis | Prednisone | UNK | Favourable |
| Eland 2023 | ♀ 25 | RRMS | IFN β, DMF | UNK | Nausea, epigastric pain, loose stools, panniculitis | 2 weeks | IBD akin to ulcerative colitis. | UNK | Ulcerative colitis | Corticosteroids | UNK | UNK |
|  | ♀ 44 | PPMS | UNK | UNK | Abdominal pain, diarrhoea | After 1^st^ dose | IBD akin to ulcerative colitis. | UNK | Ulcerative colitis | Amoxicillin / clavulanic acid | UNK | UNK |
| Garcia-Estevez 2025 | ♀ 35 | RRMS | UNK | UNK | Diarrhoea, rectal bleeding, abdominal pain | 1 year | Chronic colitis with acute activity of moderate intensity at the level of the right and transverse colon and mild activity at the level of the rectum‑sigma, compatible with idiopathic IBD | UNK | IBD | Budesonide, mesalamine, vedolizumab | Stopped, replaced by alemtuzumab | Favourable |
| Kean 2024 | ♀ 36 | MS | UNK | UNK | Diarrhoea, abdominal pain, fever | 15 months | Pancolonic ulceration | UNK | Colitis | Hydrocortisone, infliximab, azathioprine | Stopped, replaced by natalizumab | Favourable |
|  | ♀ 38 | MS | UNK | UNK | Diarrhoea, abdominal pain | 18 months | Crypt abscess formation and granulomas. | UNK | Colitis | Hydrocortisone | Stopped, replaced by natalizumab and stem cells allograft | Favourable |
|  | ♂ 32 | MS | UNK | UNK | Anorexia, diarrhoea | 2 years | UNK | UNK | Colitis | Corticosteroids, ustekinumab | Stopped, stem cells allograft | Favourable |
| Lieb 2023 | ♀ 60 | PPMS | UNK | UNK | UNK | 4 years | IBD and CMV infection | UNK | Colitis | Corticosteroids, antiviral | Stopped | Favourable |
| Lee 2020 | ♀ 43 | MS | Alemtuzumab, teriflunomide | UNK | Watery dark stools, abdominal pain | 1 year | Mucosa in proximal colon appears normal. Rest of the colonic mucosa completely ulcerated without residual islands of intact mucosa. Congestion, chronic inflammation, and submucosal fibrosis. Ulceration mostly superficial but extend focally to the muscularis propria. Focal areas of subserosal fibrosis. Pathology features compatible with clinical diagnosis of medication induced colitis. | Calprotectin ↑, CRP ↑ | Colitis | Hydrocortisone, metronidazole, oral vancomycin, colectomy | UNK | Favourable |
| Mallick 2024 | ♂ 26 | MS | UNK | UNK | Emesis | 7 months | UNK | Neutropenia | Typhlitis | UNK | UNK | UNK |
| Malloy 2022 | ♀ 40 | RRMS, coeliac disease | IFN β1a, PEG-IFN β1a, fingolimod | UNK | Chronic postprandial abdominal cramps, diarrhoea | 10 months | No active coeliac disease. patchy mild active chronic colitis in samples from the caecum and ascending colon without the presence of granulomas. | Calprotectin ↑ | Fulminant colitis | Methylprednisolone, mesalamine, mercaptopurine, ciclosporin, colectomy | Stopped, replaced by teriflunomide | Favourable |
| Shah 2022 | ♀ 42 | MS | UNK | UNK | Fever, abdominal pain, watery diarrhoea | 1 week | Diffuse active colitis with neutrophilic infiltration, crypt abscesses, and focal erosion/ulceration. No evidence of chronic inflammation, viral inclusions, amoeba, or other micro-organisms. | UNK | Enterocolitis | Methylprednisolone, prednisone, ustekinumab | Stopped | Favourable |
| Sunjaya 2020 | ♂ 47 | PPMS | DMF | UNK | Bloody diarrhoea, fever | Several weeks | Nonspecific severe chronic inflammation | UNK | Refractory colitis | Prednisone, hydrocortisone, sigmoidectomy | Stopped | Not recovered |
| Thakurdesai 2023 | ♀ 2* | MS | UNK | UNK | Non-bloody diarrhoea, abdominal tightness | 2 years (1 week) | Severe active colitis | UNK | Colitis | Prednisone, ganciclovir, loperamide | Stopped | Favourable |
| Tolaymat 2023 | ♀ 67 | RRMS | Stem cells allograft, glatiramer | UNK | Diarrhoea, occasional abdominal cramps | 32 months | UNK | Lymphopenia, CRP ↑ | Colitis | Prednisone | Stopped, replaced by teriflunomide | Favourable |
|  | ♀ 26 | RRMS, JCV infection | Glatiramer, natalizumab | UNK | Frequent diarrhoea, occasional blood in stools, fever, abdominal pain and cramps, nausea, vomiting | 1.5 year | UNK | UNK | Crohn disease | UNK | Stopped, replaced by cladribine | Favourable |
| Tuqan 2020 | ♀ 61 | MS | UNK | UNK | Recurrent diarrhoea after each injection | UNK | Drug-induced colitis | UNK | Colitis | Budesonide, mesalamine | Stopped | Favourable |
| Viti 2024 | ♀ 38 | RRMS, JCV infection, auto-immune thyroiditis, fingolimod induced lymphopenia, cholecystectomy, chronic gastritis | IFN β1a, fingolimod, corticosteroids | Levothyroxine | Watery diarrhoea, vomiting, pharyngodynia | 4 months | mucosa of the small intestine showing preserved villous architecture and minimal lymphoplasmacytic infiltrate of the lamina propria. Fragments of mucosa of the large intestine exhibiting regular glandular morphology and mucin production, presence of oedema, lymphoid aggregates, and haemorrhagic extravasation of the lamina propria. Immunohistochemical CD3 staining: no increase in intraepithelial lymphocytes in either the small or large intestine. Masson’s trichrome stain: no collagenous deposits in any of the biopsy sites. | UNK | Colitis | Beclometasone, metoclopramide, scopolamine | Stopped, replaced by ofatumumab | Favourable |
| Voda 2025 | ♀ 35 | RRMS | UNK | UNK | Abdominal pain, diarrhoea | 2 years | Focal active colitis, showing regenerative areas, microabscesses and erosions, without signs of intraepithelial dysplasia or neoplastic proliferation. | Inflammatory syndrome, Calprotectin ↑ | Colitis | Methylprednisolone, mesalamine | Stopped, replaced by natalizumab | Favourable |
|  | ♂ 26 | RRMS, JCV infection | Glatiramer | UNK | Abdominal pain, cramps, diarrhoea, bloody stools, weight loss | 1 month | Chronic active non-granulomatous colitis | Calprotectin ↑, C. difficile + | Colitis | Corticosteroids, mesalamine | Stopped, replaced by cladribine | Favourable |
|  | ♀ 37 | RRMS | UNK | UNK | Watery stools, blood streaks | 2 years | No signs of inflammation or dysplasia | UNK | Colitis | UNK | UNK | UNK |
| 5-ASA: 5-aminosalycicylic acid, AH: arterial hypertension, CRP: C-reactive protein, DMF: dimethylfumarate, GERD: gastroesophageal reflux disease, IBD: inflammatory bowel disease, IFN: interferon, JCV: John Cunningham virus, MS: multiple sclerosis, NA: not applicable, PCOS: polycystic ovary syndrome, PH: pulmonary hypertension, PPMS: primary progressive multiple sclerosis, RRMS: remitting-relapsing multiple sclerosis, TTO: time to onset, UNK: unknow, y.o.: years-old  *Probable typo in the article considering MS’s epidemiology and ocrelizumab’s indications, age excluded from calculation because of that | | | | | | | | | | | | |
